# UDP-*N*-acetyl-α-D-galactosamine:polypeptide *N*-acetylgalactosaminyl-transferase from the snail *Biomphalaria glabrata* – substrate specificity and preference of glycosylation sites

**DOI:** 10.1007/s10719-014-9565-3

**Published:** 2014-10-23

**Authors:** Christopher Taus, Markus Windwarder, Friedrich Altmann, Reingard Grabherr, Erika Staudacher

**Affiliations:** 1 Department of Chemistry, Glycobiology, University of Natural Resources and Life Sciences, Muthgasse 18, 1190 Vienna, Austria; 2 Department of Biotechnology, University of Natural Resources and Life Sciences, Muthgasse 18, 1190 Vienna, Austria; 3 Present Address: Institute of Urology, Medical University of Vienna, Währinger Gürtel 18-20, 1090 Vienna, Austria

**Keywords:** ppGalNAcT, GalNAc-transferase, O-glycosylation, *Biomphalaria glabrata*

## Abstract

O-glycosylation is a widely occurring posttranslational modification of proteins. The glycosylation status of a specific site may influence the location, activity and function of a protein. The initiating enzyme of mucin-type O-glycosylation is UDP-GalNAc:polypeptide GalNAc transferase (ppGalNAcT; EC 2.4.1.41). Using electron-transfer dissociation mass spectrometry, ppGalNAcT from the snail *Biomphalaria glabrata* was characterized regarding its ability to glycosylate threonine and serine residues in different peptide sequence environments. The preferences of the snail enzyme for flanking amino acids of the potential glycosylation site were very similar to vertebrate and insect members of the family. Acceptor sites with adjacent proline residues were highly preferred, while other residues caused less pronounced effects. No specific O-glycosylation consensus sequence was found. The results obtained from synthetic peptides were in good correlation with the observed glycosylation patterns of native peptides and with the order of attachment in a multi-glycosylated peptide. The snail enzyme clearly preferred threonine over serine in the *in vitro* assays. No significant differences of transfer speed or efficiency could be detected using a mutant of the enzyme lacking the lectin domain. This is the first characterisation of the substrate specificity of a member of the ppGalNAcT family from mollusc origin.

## Introduction

Glycosylation plays an important role in several types of recognition processes ranging from fertilization and development to pathological events and cell death. In earlier times of glycobiology the role of O-glycans was merely seen in the influence of physical parameters, like ensuring protein stability and tertiary structure, serving as “stalks” in membrane bound receptors and providing the basis for rigid conformation of the polypeptide backbone; the more specialized functions had been allocated to N-glycans. In the meantime it has been shown, that O-glycans can be also relevant for the fine tuning of biological processes, such as modulation of enzyme activity, they may act as signal molecules or sorting determinants [1–5]. Mucin-type O-glycosylation is the most frequent type of O-glycosylation, present in all kinds of animals containing an *N*-acetylgalactosamine as the linkage sugar to a hydroxy amino acid of a peptide [6–8]. This precursor is further elongated by adding galactoses, *N*-acetylglucosamines, *N*-acetylgalactosamines, fucoses, sialic acids and other sugars depending on the organism, tissue and developmental stage. The first enzyme involved in the biosynthesis of these O-glycans is a member of an evolutionarily highly conserved family of UDP-GalNAc:polypeptide GalNAc transferases (ppGalNAcTs, [EC 2.4.1.41]), which transfer the GalNAc residue from UDP-GalNAc to a serine (Ser) or threonine (Thr) of a polypeptide chain. The family members share a common structure consisting of a short transmembrane domain, a stem region, a Gal/GalNAc motif, a manganese binding site and, quite commonly, a ricin-like lectin domain. Small differences in structure seem to be related to tissue distribution and acceptor specificity.

In humans 20 representatives of this enzyme family have been found, 19 in rodents, 14 in *Drosophila melanogaster*, 9 in *Caenorhabditis elegans* and several in other organisms [9–11]. The large number of seemingly redundant homologues of the same enzyme in one organism indicates that it requires a reliable backup system. In most of the experiments in mice the loss of a single GalNAcT-gene caused no obvious phenotype (for a review see 10). However, in *Drosophila melanogaster* ppGalNAcTs have been shown to be essential for viability [12].

The ppGalNAcTs have been clustered into groups and subgroups by their primary structure [10]. While N-glycosylation is restricted to Asn-residues within the consensus sequence Asn-X-Ser/Thr, for mucin-type O-glycosylation no strict amino acid sequence can be determined. However, a number of studies on vertebrate enzymes demonstrate that the different groups and subgroups favour specific amino acids close to the glycosylation site of the acceptor peptide. Furthermore these groups differ also in their ability to transfer GalNAc-residues to already glycosylated acceptor substrates and their expression levels vary for different tissues.

Molluscs are highly successful in survival, are able to adapt to changing environments and are intermediate hosts of some parasites. They combine glycosylation features of mammals, worms and insects which makes them an interesting model for studies of biosynthetic pathways, in particular glycosylation processes. ppGalNAcT from *Biomphalaria glabrata* (GenBank: KC18251), so far the only cloned and characterized glycosyltransferase from mollusc origin, is a 600 amino acid type II membrane protein containing all the above mentioned structural domains [13]. It is a member of group Ib being a typical T2 enzyme [10], with a pH optimum at 6.0–6.5, dependence on divalent cations and it is able to glycosylate non- as well as multi-glycosylated acceptor peptides [13].

Here we present a detailed evaluation of the snail ppGalNAcT donor and acceptor preferences and elucidate the order and position of the glycosylated amino acids in case of multi-glycosylation. Furthermore, the influence of the lectin domain on the specificity of the snail enzyme is revealed.

## Material and methods

### Materials

*Spodoptera frugiperda* cells (Sf9, ATCC CRL-1711) were cultivated in IPL41 medium (SAFC Biosciences, St. Louis, USA) containing yeast extract, a lipid mixture supplemented with 10 % fetal calf serum, at 27 °C [14]. Acceptor peptides were obtained from Cellmano Biotech Co., Ltd., Shanghai, China (Table 1).

**Table 1:** Acceptor peptides used in this study

Name	Sequence
Muc1a	APPAHGVTSAPDTRPAPGC
Muc1a’	AHGVTSAPDTR (short version of Muc1a)
Muc2	PTTTPITTTTTVTPTPTPTGTQTK
Muc5Ac	GTTPSPVPTTSTTSAP
CHT 1	APPAHPGP **T** PGPRPAPG^a^
CHT 2	APPAHPGV **T** PGPRPAPG
CHT 3	APPAHPGP **T** PGYRPAPG
CHT 4	APPAHPGP **T** PGKRPAPG
CHT 5	APPAHPGP **T** PGHRPAPG
CHT 6	APPAHPGV **T** PGYRPAPG
CHT 7	APPAHPGI **T** PGPRPAPG
CHT 8	APPAHPGF **T** PGPRPAPG
CHT 9	APPAHPGP **T** EGPRPAPG
CHT 10	APPAHPGP **T** IGPRPAPG
CHT 11	APPAHPGP **T** PRPRPAPG
CHT 12	APPAHHGP **T** PGPRPAPG
CHT 13	APPAHPVP **T** PGPRPAPG
CHT 14	APPAHPGL **T** PGPRPAPG
CHT 15	APPAHHVV **T** ERYRPAPG
CHT 16	APPAHDFV **T** PAPRPAPG
CHT 17	APPAHDFP **T** PAPRPAPG
CHT 18	APPAHPFV **T** PAPRPAPG
CHT 19	APPAHIFV **T** PAPRPAPG
CHT 20	APPAHVFV **T** PAPRPAPG
CHT 21	APPAHDGV **T** PAPRPAPG
CHT 22	APPAHPGP **T** PAPRPAPG
CHT 23	PSSSPISSSSSVSPSPSPSGSQSK (analog of Muc2 with Thr changed to Ser)
CHT 24	APPAHGVSTAPDTRPAPGC (analog of Muc1a with an interchange of Thr and Ser)

^a^For clarity in the synthetic peptides CHT 1 – CHT 22 the Thr residue is bold and the amino acid residues which are varied are underlined

### Expression of ppGalNAcT from Biomphalaria glabrata

The enzyme, ppGalNAcT, without cytoplasmic tail and transmembrane domain (amino acid 1–26) was expressed and purified exactly as in [13]. A further truncated version, ΔppGalNAcT, omitting the complete lectin domain (lacking amino acid 477–600) was obtained in the same way. Protein concentrations were determined by the Micro-BCA protein assay (Pierce, Bonn, Germany) with bovine serum albumin as the standard.

### ppGalNAcT activity assay

The enzyme activity of ppGalNAcT and ΔppGalNAcT was determined in 20 μl reaction mixture containing 50 mM MES (2-(*N*-morpholino)ethanesulfonic acid), pH 7.0, 10 mM MnCl_2_, 2 mM UDP-GalNAc (Sigma-Aldrich, Vienna, Austria), 10 nmol acceptor peptide (Cellmano Biotech Co., Ltd., Shanghai, China) and 2 μl enzyme solution (ppGalNAcT or ΔppGalNAcT, protein concentrations 5.0 μg/ml) at 37 °C for 90 min.

For determination of donor specificity UDP-Gal, UDP-GlcNAc, GDP-fucose, UDP-xylose, UDP-glucuronic acid (all from Sigma-Aldrich, Vienna, Austria) and CMP-neuraminic acid (Jennewein Biotechnologie GmBH, Germany) were used instead of UDP-GalNAc. For time courses the standard assay was conducted for 30 h and aliquots were taken at 1, 2, 4, 6, 8, 23, 26 and 30 h. For determination of the position of the transferred GalNAc-residues within the peptide, incubation was carried out with Muc1a, Muc1a’, Muc5Ac and Muc2, respectively (for sequences see Table 1), for 24 h analogously to the standard assay with additions of 10 nmol UDP-GalNAc and 2 μl of enzyme solution after 4, 8 and 20 h.

Each assay was carried out at least in duplicate with appropriate controls.

### Analysis of enzyme activity

Quantitative analysis was performed by HPLC on a reversed phase C18 column (4.6 × 250 mm, 5 μm, Thermo Scientific, Vienna, Austria) in 0.5 % trifluoroacetic acid in water, applying a linear gradient from 15 to 25 % of eluent (0,1 % trifluoroacetic acid in acetonitrile) in 20 min at a flow rate of 1 ml/min with a detection at 220 nm. All quantitative values were calculated from the area of HPLC patterns.

### Preparation of mono-glycosylated acceptors

Mono-glycosylated Muc2 and Muc5Ac were obtained using the standard assay conditions with 2 h of incubation time, followed by preparative runs on HPLC exactly as for the quantification analysis. The peak containing the mono-glycosylated peptide was collected manually.

### Matrix assisted laser desorption ionisation – time of flight (MALDI-TOF) mass spectrometry

MALDI-TOF MS analysis was carried out on an Autoflex Speed MALDI-TOF (Bruker Daltonics, Germany) equipped with a 1000 Hz Smartbeam.II laser in positive mode using α-cyano-4-hydroxycinnamic acid as matrix (1 % in 65 % acetonitrile solution). For crystallization 1 μl of an 1:40 dilution of the samples was spotted on the plate, air dried, covered by 1 μl of matrix solution and again air dried. Spectra were processed with the manufacturer’s software (Bruker Flexanalysis 3.3.80).

### Electron Transfer Dissociation (ETD) mass spectrometry

ppGalNAcT activity assay solutions were purified *via* C-18 SPE cartridges (25 mg, Thermo Scientific). Briefly, the cartridges were equilibrated with 500 μl methanol, 500 μl 65 % acetonitrile and washed twice with 500 μl 0,1 % formic acid. Sample was applied, washed twice with 500 μl 0,1 % formic acid and Muc peptide was eluted with 65 % acetonitrile. For direct infusion ETD-MS, the samples were vacuum-dried and redissolved in 50 % acetonitrile.

Muc peptides were directly infused into a Bruker amaZon speed ETD ion trap using a Hamilton syringe at a flow rate of 2 μl/min. The mass spectrometer was operated in Manual MS(n) mode with ETD as fragmentation mode. Triply or four times charged precursors ions were measured with the following settings: ICC target 200000, maximum accumulation time 10 ms, isolation width 4 amu, ETD reagent time 60–100 ms. Data were recorded for about 10–15 min for every glycopeptide form and analysed with Bruker’s Data Analysis 4.0. Average mass spectra were generated using the SNAP peak finder algorithm and exported to Bruker’s BioTools 3.2. *Via* the Sequence Editor, Muc peptide sequences with additional GalNAc modification(s) on every possible site (Ser or Thr) were generated and sent to BioTools. Theoretical peptide fragment masses (c- and z-type ions) were calculated for every possible glycopeptide form and compared to measured fragment masses. The identification of fragment ions, specific for a certain GalNAc modification site enabled us to illuminate the different glycosylation sites on the different Muc peptide species [15, 16].

## Results

### Determination of donor specificity

The donor specificity of snail ppGalNAcT was investigated using standard assay conditions with the acceptor substrates Muc2 and Muc5Ac peptide as well as the corresponding mono-glycosylated peptides and the nucleotide sugars UDP-Gal, UDP-GlcNAc, GDP-fucose, UDP-xylose, UDP-glucuronic acid and CMP-neuraminic acid instead of UDP-GalNAc. In none of these experiments any addition of a sugar was observed by MALDI (data not shown). Obviously, the snail enzyme is strictly specific to UDP-GalNAc. In contrast, the human ppGalNAcT2 has been found to utilize also UDP-Gal [17].

### Localisation of GalNAc residues

Previously we have shown by MALDI-TOF analysis that the snail ppGalNAcT transfers up to eight GalNAc residues to Muc2 peptide [13]. In order to obtain more information about the sites and a possible order of glycosylation, we analysed a large scale incubation of Muc2 with ESI-ETD-MS/MS. Muc2 forms with 1–8 GalNAc residues were selected as precursors for the ETD reaction and analysed consecutively. Due to the heterogeneity of the higher glycosylated Muc2 forms (especially the one with 5 GalNAcs), conclusion only about the major glycoform could be drawn. Nevertheless, an order of glycosylation of the different sites of modification by ppGalNAcT could be deduced.

**Fig. 1:**
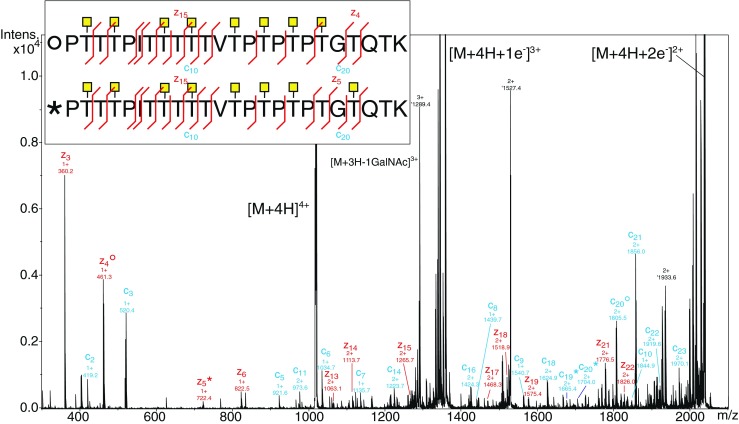
Annotated ETD-MS/MS spectrum. Muc2 peptide with eight GalNAc residues [M+4H]^4+^ at 1018 m/z was taken as a precursor for the ETD reaction. Analysis of the fragment ion masses revealed the amino acids carrying a GalNAc modification. Two different forms (indicated with _°_ and _*_), slightly differing in modification site of the most C-terminal GalNAc were identified (see insert). Fragment ions marked with _°_ or _*_ enabled the differentiation between the two glycopeptide forms

Thr-15 was clearly identified as the transferase’s first choice. With three Pro residues at positions −1, +1 and +3, this glycosylation site displays the previously published prerequisites for an ideal O-glycosylation site [18] (“+” or “−” refers to residues that are N-terminal or C-terminal to a hydroxyl amino acid. The number reflects the distance from the hydroxyl amino acid). The second GalNAc-residue was transferred to Thr-2 or Thr-13. Both have two Pro residues in close proximity. For Thr-2 they are in position −1 and +3, whereas for Thr-13 Pro is located at +1 and +3. Thr-17 with Pro in −1 and +1 is the subsequent glycosylation site, almost equal to Thr-10, which has no Pro anywhere close. Thr-19, with two Pro at −1 and −3, but none on the other side, is next target, followed by Thr-8 and Thr-4 (Table 2, Fig. 1). Glycosylation at Thr-21 was detected in a low amount and only in the form with 8 GalNAc residues, and only when no GalNAc was present linked to Thr-19. Thus, even with 14 Thr residues present in Muc2, only 9 potential glycosylation sites were identified, of which a maximum of 8 were in use, since Thr-19 and Thr-21 were never glycosylated in the same peptide (Fig. 1). GalNAc residues on directly adjacent residues were never detected. Glycosylation sites were always separated by at least one amino acid.

**Table 2:** Ranking of Thr residues in Muc2 due to their glycosylation feasibility

Rank	Thr residue
1	T-15
2	T-2, T-13
3	T-10, T-17
4	T-19
5	T-8
6	T-4
7	T-21

Also in Muc5Ac, Muc1a and Muc1a’ (which is a shorter version of Muc1a) the glycosylation sites where determined. In the sequence of Muc5Ac Ser-5 was the preferred acceptor site probably due to surrounding Pro residues at position −1, +1 and +3. With longer incubation times up to four GalNAc residues could be attached. For Muc1 and Muc1a’ only the Thr residues were identified as possible glycosylation sites (Thr-8 and Thr-5 respectively). No GalNAc attachment to Ser could be observed. After an interchange of Thr and Ser in the sequence of Muc1a (Thr-8 →Ser-8 and Ser-9 →Thr-9), the peptide (CHT 24) was no longer an acceptor for snail ppGalNAcT. CHT 23, an analogue of Muc2 with all Thr residues changed to Ser residues, had a drastically reduced GalNAc transfer rate. While on Muc2 peptide 8 GalNAc residues could easily be attached, only four GalNAc residues could be transferred to CHT 23, even after the longest incubation time.

### Determination of acceptor specificity with synthetic peptides

**Fig. 2:**
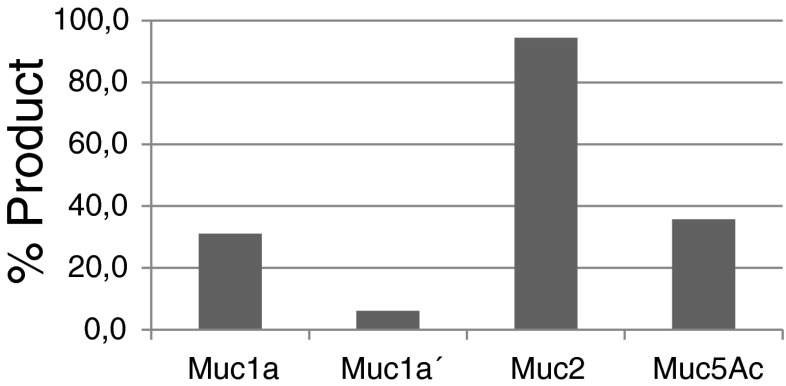
Incorporation rate of GalNAc residues into native acceptor peptides

In order to investigate the optimal amino acid sequence close to a potential O-glycosylation site, 22 synthetic variants of a good acceptor peptide with one single Thr and no Ser were tested (Table 1). The model acceptor peptide was designed based on earlier studies of human ppGalNAcT2 [18–21]. Our variation study included only three amino acids upstream and downstream from the glycosylation site, still the length of the chosen peptide was 17 amino acids in total, in order to avoid activity reductions due to too small chains. About 30 % of Muc1a peptide (19 amino acids) was converted into GalNAc-Muc1a in the standard incubation assay, whereas Muc1a’ peptide, a shorter version of Muc1a with 11 amino acids, was converted to only 6 % (Fig. 2).

The central amino acid of the model peptide was a Thr surrounded by four Pro residues in position −3, −1, +1 and +3 and two Gly at −2 and +2. These Pro and Gly residues were changed, while the five amino acids at the N- and at the C-terminus, respectively, were kept the same for all peptides. Glycosylated products of the standard incubation assay were analysed by MALDI-TOF MS and quantified by HPLC. The acceptor peptides were sorted into 4 groups according to acceptor quality (Table 3): group 1 – complete glycosylation within the time of the standard assay; group 2 – over 50 %; group 3 – 5–49 %; and group 4 - only traces were glycosylated. For those peptides which were completely converted within the standard conditions (CHT 1, CHT 3, CHT 9, CHT 10, CHT 11, CHT 12, CHT 13), the experiment was repeated with a quarter of the previous amount of enzyme. This revealed that for three of them (CHT 3, CHT 9, CHT 12) the change of one Pro (position −1: Pro→His; +1: Pro→Glu; +3: Pro→Tyr) enhanced the quality of the acceptor, all the others slightly reduced incorporation compared with CHT 1. Changing the Gly in position +2 to Arg (CHT 11) did not affect the incorporation while the change to another basic amino acid (His) led to a drastic decrease (CHT 5). Only a moderate decrease was seen changing at −2 Gly→Val (CHT 13) or at −1 Pro→Val (CHT 2). Combining all changes with moderate effect (His-Val-Val-Thr-Glu-Arg-Tyr, CHT 15), activity was completely abolished.

**Table 3:**
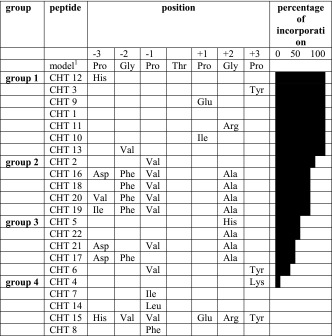
Peptides listed by their quality as acceptors for snail ppGalNAcT. For clarity only those amino acids are given that are changed compared to the model peptide. Group 1: very good acceptors (95–100% of acceptor conversion using standard conditions), group 2 moderate acceptors (50–94% conversion), group 3: weak acceptors (5–49% conversion), group 4: incorporation less than 5%

^a^Ideal acceptor peptide for vertebrate ppGalNAcT2 according to [18]

A similar example for a poor combination is CHT 6. Changing both Pro to Tyr at +3, which alone increased activity (CHT 3), and Pro to Val at −1, which alone only moderately decreased activity (CHT 2) resulted in a significant decrease of acceptor quality (CHT 6).

But also an increase of activity could be observed by combining some changes that decreased acceptor activity previously: a change of Gly→Ala at +2 gives a rather weak acceptor (CHT 22). But this change together with Gly→Phe at −2 and Pro→Val at −1 (CHT 18) increased acceptor quality. If there was an additional change of Pro→Asp at −3 a further activity increase was detected (CHT 16) while Pro→Val or Ile at −3 had a less pronounced effect (CHT 20, CHT 19), yet, was still better than Ala alone (CHT 22). When the Pro→Asp change at −3 was combined with Ala at +2 and Phe at −2 OR Val at −1 (CHT 17, CHT 21), the activity was less than for Ala alone (CHT 22).

The −1 position was found to be especially sensitive to changes. There, Pro→Val yielded in moderate reduction (CHT 2) while Pro→Ile, Leu or Phe resulted in a strong decrease (CHT 7, CHT 14) or complete abolishment of activity (CHT 8). However, the change of Pro to Ile at +1 resulted only in a slightly weaker acceptor quality (CHT 10).

Taken together, the results obtained with the synthetic peptides, Pro residues in −3, −1, +1 and +3 favoured GalNAc addition, but other amino acids may be also present (−3: His, Asp; −2: Val, Phe; −1: Val; +1: Glu, Ile; +2: Arg, Ala, His; +3: Tyr). There seems to be no strict requirement for a certain amino acid at a specific position except that some amino acids completely prevented transfer (−1: Ile, Leu, Phe; +3: Lys). Generally, we found that the more amino acids we changed in the acceptor peptide that were previously identified as “ideal” for vertebrates, the less efficient the acceptor became.

### Evaluation of glycosylation sites of native acceptors

ETD-MS/MS, which had been already applied by Yoshimura [22] to identify the glycosylation sequence carried out by human ppGalNAcT3 on Muc5Ac, was used for revealing the incorporation of GalNAc into the native acceptors Muc1a, Muc1a’, Muc2 and Muc5Ac. The preferred glycosylation sites correlated well with the preferences shown above. Thr-8 of Muc1a is flanked by the sequence His-Gly-Val upstream and Ser-Ala-Pro downstream, which is for sure a good variant. In contrast, Thr-13 is located between Ala-Pro-Asp and Arg-Pro-Ala, which is not surprisingly an unfavourable combination.

Shortening of the total chain (Muc1a’) did not change the preference but reduced the transfer rate. Changing the Thr to Ser in the previously preferred glycosylation site of this peptide abolished its acceptor qualities totally (CHT 24). This is surprising as a Ser residue in a similar good neighbourhood Thr-Thr-Pro-Ser-Pro-Val-Pro is the enzyme’s first choice in Muc5Ac.

The first sites of incorporation into Muc2 can be predicted by the surrounding environment. Thr-15 with Pro in −1, +1, and +3 was the preferred acceptor site followed by Thr residues with less Pro residues in ideal positions (see above). The weakest acceptor was Thr-21 with only one Pro at −3.

An already glycosylated Thr in −2 position or in +2 position seemed to enhance further glycosylation. This was not the case for Thr-21. A previous glycosylation of Thr-19 prohibited any glycosylation at Thr-21 as we never found glycosylated products on both of these sites.

A big exception to all these empirical rules was the fast glycosylation of Thr-10 despite the lack of Pro in close neighbourhood. There is a Val at +2 and an already glycosylated Thr at +3; the rest are not glycosylated Thr residues. The already glycosylated Thr may be the reason of the conversion of the sequence into an attractive acceptor.

### Influence of the lectin domain on substrate specificity

All native and synthetic peptides were also tested using an enzyme variant devoid of the lectin domain (ΔppGalNAcT). Overall the specificity for the synthetic acceptors with one glycosylation site was similar. No differences were observed for group 1 and group 4. In group 2 and 3 a change in −2 position had more influence on the activity towards an acceptor without lectin domain than on the complete enzyme. CHT 13 with Gly→Val in position −2 turned out to be a weaker acceptor for ΔppGalNAcT. Only 46 % incorporation was detected by the enzyme without lectin domain compared to 100 % transfer by the enzyme containing the lectin domain. While Phe at that position often slightly increased the transfer (CHT 16: 70→80 %, CHT 18: 70→75 %).

**Fig. 3:**
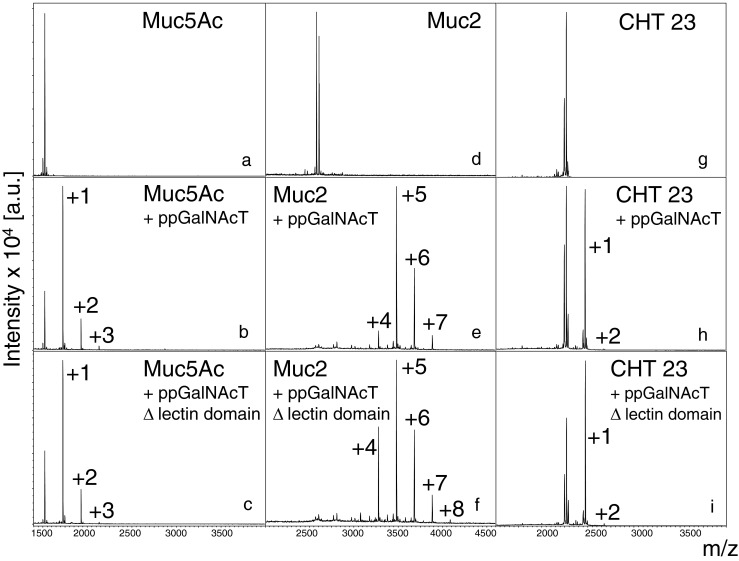
MALDI-TOF ([M+ Na]^+^) analysis of the transfer of GalNAc residues using Muc5Ac, Muc2 or CHT 23 as acceptor peptide. Acceptor peptide without incubation (a,d,g); 8-h-incubation with ppGalNAcT containing the lectin domain (b,e,h); 8-h-incubation with ppGalNAc without the lectin domain (c,f,i). The numbers indicate the incorporated GalNAc residues

For the native substrates Muc2 and Muc5Ac we performed a time course with the complete enzyme and with ΔppGalNAcT. Samples were analysed by HPLC and MALDI-TOF MS after 1, 2, 4, 6, 8, 23, 26 and 30 h. Both enzyme variants incorporated the same number of GalNAc residues (maximum 8 in Muc2 and 4 in Muc5Ac) and exhibited the same incorporation rate. A similar result was obtained for CHT 23 (maximum 4 GalNAc residues)., which is the Ser-version of Muc2. Remarkably, there was no statistical significant difference in the transfer rate comparing the enzyme variants with and without lectin domain (Fig. 3).

## Discussion and conclusion

Despite a considerable phylogenetic distance, the recently identified ppGalNAcT from the snail *Biomphalaria glabrata* displays homology to human ppGalNAcT, especially to ppGalNAcT2 (74 % positives with 58 % identity according to NCBI blast analysis). Here, we aimed at a characterization of the donor and acceptor substrate specificity to find out about parallels or differences between snail ppGalNAcT and human ppGalNAcT2, which is the only crystallised [37] and the best characterised member of the ppGalNAcT2 homologs.

The core regions of our synthetic peptides used in this study were variants of Pro-Gly-Pro-Thr-Pro-Gly-Pro, which had been identified by Gerken *et al*. to be an ideal acceptor peptide for human ppGalNAcT2 [18]. In order to overcome the activity reduction seen with too small chains we added 5 amino acids on both ends of this core, resulting in peptides containing 17 amino acids.

In previous studies on vertebrate ppGalNAcT2 and on the *Drosophila* ortholog PGANT2 the enhancing activity of Pro residues close to the glycosylation site had been determined [19, 23–25]. Especially the −1 position was found to be particularly relevant [20]. Charged amino acids in general decreased the activity, while Ser, Thr, Ala and Gly residues seemed to be acceptable in most of the positions. Neither strong hydrophobic nor hydrophilic amino acids seemed to be necessary. Large, especially aromatic side chains abolished activity [19, 20, 24, 26]. On the basis of the evaluation of a large set of O-glycosylation sites of vertebrate proteins, Elhammer *et al*. developed an algorithm for the prediction of probable glycosylation sites. The accuracy of the method is about 75–85 % [19, 27].

In general, the results found for vertebrate ppGalNAcTs correlate with the data we received for the *Biomphalaria glabrata* enzyme [28–31]. Only small differences were detected. At +2 position Gly and Ala had been identified as the most favourable amino acids for the human ppGalNAcT2 and an *in vivo* system using COS7 cells [18, 20]. The snail enzyme clearly preferred Gly and Arg but was less in favour for Ala in this position. An enhancement by Tyr at +3 was found for human ppGalNAcT1, but not for human ppGalNAcT2 or *Drosophila* PGANT2 [18, 25], albeit snail ppGalNAcT2 activity was elevated by Tyr at +3. Similar to human ppGalNAcT2 Phe, Ile, Leu decreased activity significantly but Val was not that bad for the snail enzyme as it had been described for the human one [18]. Similar to human ppGalNAcT2 Pro at −1 and +3 was very important, while Glu at +1 was less supportive for the human than for the snail enzyme. His at −3 prevented activity of human ppGalNAcT2, however, the snail enzyme was enhanced by this modification. Lys at +3 was inhibitory for snail ppGalNAcT2.

Studying the order of O-glycosylation of Muc5Ac, we found that the snail ppGalNAcT2 used Ser-5 rather than Thr-9 as the first glycosylation site which is in contrast to human ppGalNAcT2 [31]. This is surprising, as in all other cases the snail enzyme clearly preferred Thr over Ser residues, which is in accordance with other ppGalNAcT2s [17, 19]. However, an early study on ppGalNAcTs using a preparation from bovine colostrum found that *in vivo* the preference may be different as compared to the *in vitro* requirements [26].

As soon as the acceptors were already glycosylated, the situation became even more complex. While the above mentioned preferences for neighbouring amino acids were still visible, the glycosylation status of adjacent Thr residues influenced the choice of attachment sites. Similar to the human ppGalNAcT2 the snail enzyme did not require previous glycosylation [31]. We also did not observe a preference for C-terminally placed GalNAc-O-Thr residues as has been shown for human ppGalNAcT2 [32]. In agreement with earlier results from vertebrates [33–36], glycosylation of potential glycosylation sites next to an already occupied site was not detected. Therefore, the snail enzyme is definitely not a “finishing” enzyme like human ppGalNAcT7, ppGalNAcT10 or ppGaNAcT15 which add the final GalNAc residues to already highly glycosylated acceptors, even in neighbouring positions. Thus, one may speculate that the snail genome harbours further ppGalNAcT homologs.

In comparison with all other so far analysed ppGalNAcT2s from vertebrates and also from *Drosophila melanogaster*, the enzyme from *Biomphalaria glabrata* displays similar preferences for flanking amino acids of a potential glycosylation site. It has a broad specificity for non-glycosylated as well as glycosylated acceptor substrates. Thr is preferred over Ser. but predictions about glycosylation of a specific site must be made with caution.

Several studies have been performed discussing the influence of the lectin domain on the glycosylation behaviour. It was shown that the lectin domain was not necessary for catalytic activity of human ppGalNAcT2 but shifts in the preferred sites of glycosylation and higher densities of glycosylation may occur [30–32, 37, 38]. For the snail enzyme we did not observe remarkable differences of transfer for acceptors with one single glycosylation site as well as for acceptors with multiple glycosylation sites, neither in speed of incorporation nor in the final number of attached GalNAc residues.

Taken together, this first thorough study of the substrate specificity of a ppGalNAcT from a snail reveals remarkable similarities to the transferases from vertebrates and insects. One can speculate that also molluscs contain more than one type of ppGalNAcT, like it has been shown for the other so far analysed species. Considering several millions of years of separate evolution, this high degree of functional conservation strongly suggests a highly important common function or set of functions of protein O-glycosylation.

## Abbreviations

ETD: Electron Transfer Dissociation
Gal: Galactose
GalNAc: *N*-acetylgalactosamine
Glc: Glucose
GlcNAc: *N*-acetylglucosamine
MALDI-TOF: Matrix assisted laser desorption ionisation – time of flight
MES: 2-(*N*-morpholino)ethanesulphonic acid
MS: Mass spectrometry
ppGalNAcT: polypeptide *N*-acetylgalactosaminyltransferase
Sf9-cells: *Spodoptera frugiperda* cells
